# Fabrication of BiOBr_x_I_1−x_ photocatalysts with tunable visible light catalytic activity by modulating band structures

**DOI:** 10.1038/srep22800

**Published:** 2016-03-07

**Authors:** Xing Zhang, Chu-Ya Wang, Li-Wei Wang, Gui-Xiang Huang, Wei-Kang Wang, Han-Qing Yu

**Affiliations:** 1 CAS Key Laboratory of Urban Pollutant Conversion, Department of Chemistry, University of Science & Technology of China, Hefei, 230026, China

## Abstract

A series of BiOBr_x_I_1−x_ solid solutions were explored as novel visible light-sensitive photocatalysts. These BiOBr_x_I_1−x_ solid-solution photocatalysts grew into two-dimensional nanoplates with exposed (001) facets and possessed continuously modulated band gaps from 2.87 to 1.89 eV by decreasing the Br/I ratio. The photocatalytic activities of these samples were measured, and the samples exhibited visible light-driven activities for the degradation of Rhodamine B (RhB). In particular, BiOBr_0.8_I_0.2_ exhibited the highest activity for the degradation of RhB. This result could be attributed to the balance between the effective light absorption and adequate redox potential. Additionally, investigations into the photocatalytic mechanism showed that the photodegradation of RhB over BiOBr_0.8_I_0.2_ solid-solution photocatalysts involved direct holes oxidation, in which the reaction that dominated during photocatalysis was determined by the potential of the valence band. Furthermore, a high stability in the photocatalytic activity of BiOBr_0.8_I_0.2_ was demonstrated by the cycling photocatalytic experiment and long-term irradiation, which might offer opportunities for its practical application as a catalyst.

Semiconductor photocatalysis has inspired intensive interests in recent years because of its promising applications in energy generation and environmental purification^1^. A high performance photocatalyst requires efficient absorption of visible light^2^, effective separation of photogenerated carriers, and a sufficient reduction or oxidation potential^3^. Attempts have been made to improve the photocatalytic efficiencies of photocatalysts, such as, homo/hetero-junction^4^,^5^,^6^,^7^,^8^,^9^, phase-junction^10^,^11^, solid solution^12^,^13^,^14^,^15^, facets engineering^16^,^17^, and doping^18^,^19^. Among these, the construction of a semiconductor solid solution has attracted considerable attention due to its perfectly efficient absorption of visible light via tuning the band gap of a semiconductor^20^,^21^,^22^. For example, (AgIn)_x_Zn_2(1−x)_S_2_ solid solution is an active photocatalyst for H_2_ evolution under visible light irradiation even though it is a solid solution between AgInS_2_ and ZnS and hardly possesses any activity under visible light irradiation^23^. The modified (Ga_1−x_Zn_x_)(N_1−x_O_x_) solid solution could also be modified to extend the absorption edge to longer wavelengths, although neither GaN nor ZnO absorbs visible light^24^.

Recently, bismuth oxyhalides have received extensive interests as photocatalysts due to their unique layered structures and indirect transition band gap characteristics^25^,^26^. More specifically, the layered structure consists of [Bi_2_O_2_]^2+^ slabs interleaved by double slabs of halogen ions^27^,^28^. Thus, the typical layered structure of bismuth oxyhalides are favorable for inducing an internal electric field to promote the separation of electron-hole pairs along the [001] direction and excellent photocatalytic activity^29^,^30^. For example, BiOCl nanoplates exposing (001) facets with a higher surface energy exhibited a greater catalytic activity than BiOCl nanoplates exposing (010) facets with a lower surface energy only under ultraviolet (UV)^31^. BiOI single-crystal nanosheets exhibit a higher photoactivity (approximately 7 times) than irregular BiOI for degradation of Rhodamine B (RhB) under visible light irradiation^28^. Hence, the synthesis of bismuth oxyhalide photocatalysts with exposed (001) facets has become a research focus^32^,^33^,^34^,^35^.

Among bismuth oxyhalides photocatalysts, both BiOBr and BiOI are visible light photocatalysts with band gaps of 2.87 and 1.89 eV, respectively. However, BiOBr can only absorb a very small portion of visible light^13^. As for BiOI, though its band gap is sufficiently small to cover most of the visible light range, its redox capability is limited because of the high level of the conduction band (CB)^36^. Thus, to sensitize the BiOBr photocatalyst into the visible region, the band gap could be narrowed to the level of the CB, expanded to the level of the valence band (VB), or both, which generally suppresses the redox potential. To sensitize the BiOI photocatalyst redox capability, the band gap could be widened by increasing the level of the CB, or decreasing the level of the VB, or both, which generally suppresses the visible light absorption. Accordingly, this inevitably causes an implicit contradiction between the wide visible light absorption and the adequate redox capability. In this sense, modulation of the electronic structure to achieve an optimized balance between light absorption and redox potential is a feasible approach to minimize this contradiction. Thus, the fabrication of BiOBr_x_I_1−x_ solid-solution photocatalysts with modulating band gaps and exposing active facets has emerged as a requirement.

In this work, BiOBr_x_I_1−x_ nanoplate solid solutions with tunable visible light photoactivity achieved by modulating band structures were prepared using a simple and facile solvothermal method. Their photoactivity was evaluated via the photocatalytic degradation of RhB under visible light. On the basis of the calculated energy band positions, the mechanism for the enhanced photocatalytic activity for the BiOBr_x_I_1−x_ nanoplates was elucidated.

## Results

### Phase structures and morphologies of BiOBr_x_I_1−x_

**Figure 1:**
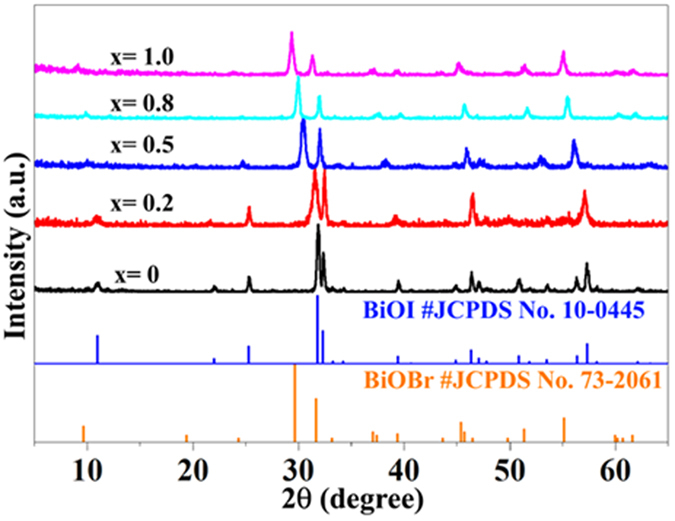
XRD patterns of the prepared samples with different values of x (x = 1.0, 0.8, 0.5, 0.2, and 0).

X-ray powder diffraction (XRD) was used to investigate the crystal phase of the prepared samples. Figure 1 shows the XRD patterns of the samples with different x values (x = 1.0, 0.8, 0.5, 0.2, and 0), together with the standard diffraction patterns of tetragonal BiOBr (JCPDS Card No. 73-2061) and tetragonal BiOI (JCPDS Card No. 10-0445). It can be seen that the diffraction peaks of the sample where x = 1.0 and 0 are well indexed as tetragonal BiOBr and BiOI, respectively. When a certain amount of Br was substituted by I in the BiOBr crystal (x = 0.8, 0.5, and 0.2 samples), the diffraction peaks of BiOBr exhibited an obvious shift toward larger angles. The gradual shift of the XRD angle as a function of I ion content indicates that the BiOBr_x_I_1−x_ samples were solid solutions^21^.

**Figure 2:**
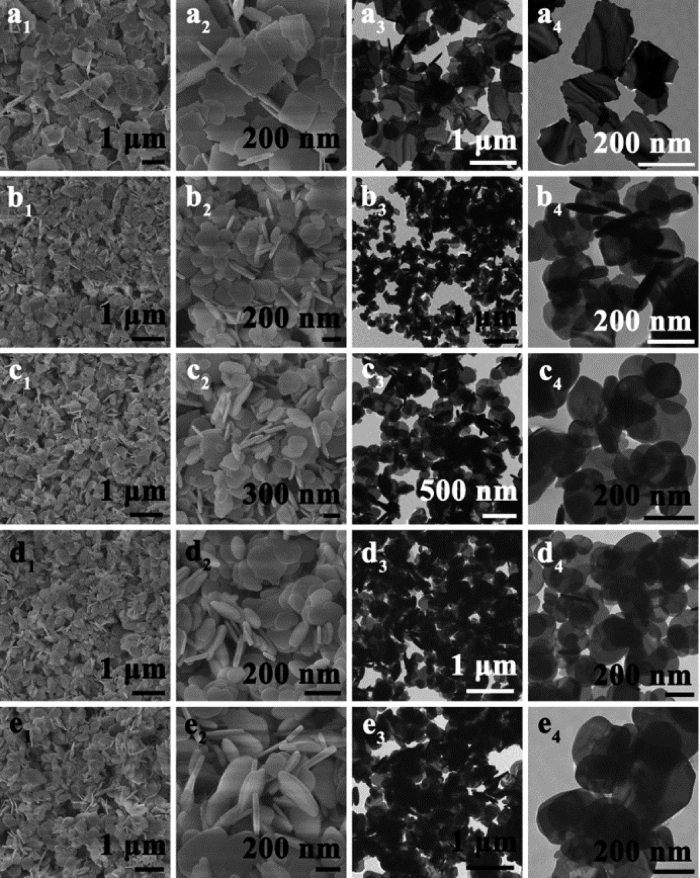
SEM and TEM images of BiOBr_x_I_1−x_ nanoplates with different values of x: (a_1_–a_4_) 1.0, (b_1_–b_4_) 0.8, (c_1_–c_4_) 0.5, (d_1_–d_4_) 0.2, and (e_1_–e_4_) 0.

The morphology and size of the samples were studied by scanning electron microscopy (SEM) and transmission electron microscopy (TEM). Figure 2a1–a2,2e1–e2 shows the large regular plates of BiOBr and BiOI, respectively, with sizes of 200–500 nm and thicknesses of approximately 15 nm. To obtain a better understanding of the BiOBr and BiOI nanoplates, TEM images of BiOBr and BiOI were obtained, as are displayed in Fig. 2a3–a4,2e3–e4, respectively, which further confirm that the obtained samples have sizes of approximately 200–500 nm. The SEM and TEM images of BiOBr_x_I_1−x_ are shown in Fig. 2b1–4–2d1–4, with x = 0.8, 0.5, and 0.2. Plate-like morphologies and similar sizes were observed. Thus, uniformly distributed BiOBr_x_I_1−x_ nanoplate solid solutions could be obtained readily by the solvothermal method.

**Figure 3:**
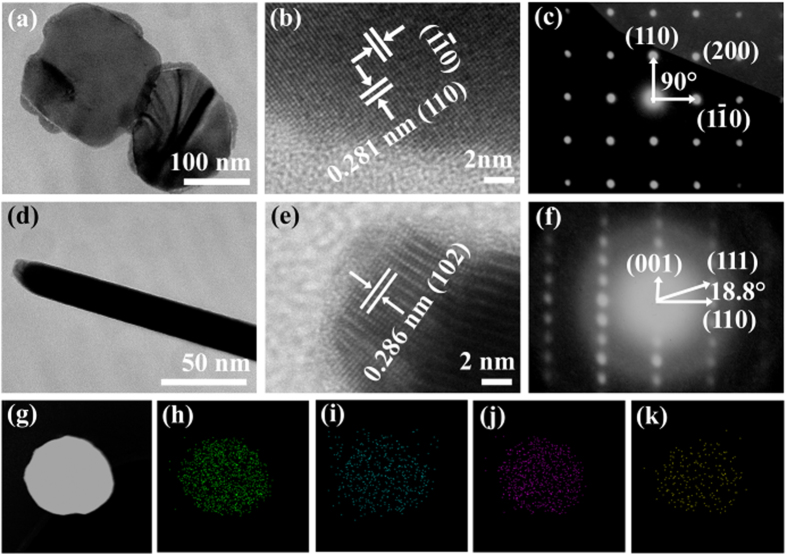
(**a,d**) TEM, (**b,e**) HRTEM images, and (**c,f**) SAED patterns of BiOBr_x_I_1−x_ nanoplates with x = 0.8, (**g**) STEM image of a single BiOBr_x_I_1−x_ nanoplate, and (**h–k**) the corresponding elemental mappings of Bi, O, Br, and I elements, respectively.

More details of the crystal structural information of the BiOBr_x_I_1−x_ nanoplates (x = 0.8) as representatives were demonstrated by high-resolution transmission electron microscopy (HRTEM). The top-view HRTEM image of the BiOBr_x_I_1−x_ nanoplate with x = 0.8 (Fig. 3b), which was taken from the edge of a single nanoplate in Fig. 3a, reveals highly crystalline and clear lattice fringes with an interplanar lattice spacing of 0.281 nm and an angle of 90° that matches well with the (110) atomic planes of the BiOBr_x_I_1−x_ nanoplate. The corresponding selected area electron diffraction (SAED) pattern (Fig. 3c) indicates a single-crystalline nature of the BiOBr_x_I_1−x_ nanoplate (x = 0.8). The angle labelled in the SAED pattern was 45°, which is consistent with the theoretical calculation of the angle between the (110) and (200) planes. The set of diffraction spots can be indexed as the [001] zone axis of the BiOBr_x_I_1−x_ nanoplate.

The side-view HRTEM image (Fig. 3e), which was taken from the tip of a lateral nanoplate in Fig. 3d, also reveals high crystallinity of the sample. The continuous lattice fringes with an interplanar lattice spacing of ~0.286 nm matched well with the (102) atomic planes of the BiOBr_x_I_1−x_ nanoplate with x = 0.8. The corresponding SAED pattern (Fig. 3f) also confirms the presence of a single crystalline BiOBr_x_I_1−x_ nanoplate. The angle labelled in the SAED pattern was 18.8°, which is consistent with the theoretical calculation of the angle between the (110) and (111) planes. The set of diffraction spots could be indexed as the [1–10] zone axis of BiOBr_x_I_1−x_ nanoplate with x = 0.8.

To evaluate the elemental composition and distribution in the BiOBr_x_I_1−x_ nanoplate, element mapping was conducted by taking a BiOBr_0.8_I_0.2_ nanoplate as a typical study target. As shown in Fig. 3h–k, it was clear that the distributions of Bi, O, Br, and I in a single BiOBr_0.8_I_0.2_ nanoplate were highly uniform, confirming the formation of the homogeneous solid solution.

### Chemical compositions and oxidation states of BiOBr_x_I_1−x_

**Figure 4:**
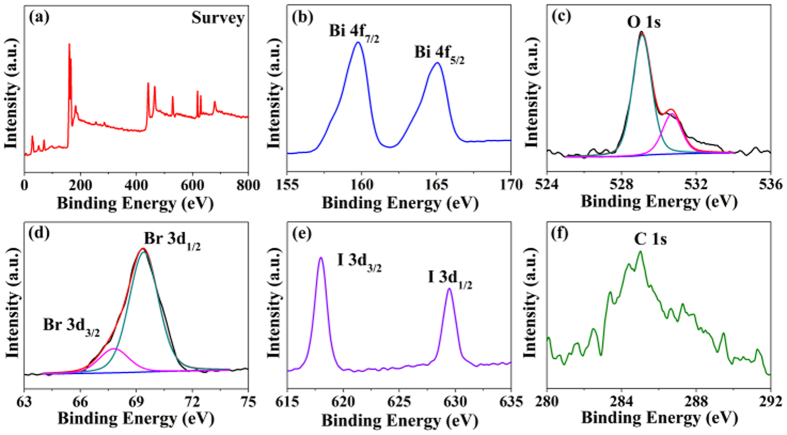
XPS spectra of the BiOBr_x_I_1−x_ sample with x = 0.8: (**a**) survey scan, (**b**) Bi 4f, (**c**) O 1s, (**d**) Br 3d, (**e**) I 3d, and (**f**) C 1s.

The chemical compositions and oxidation states of the BiOBr_x_I_1−x_ (x = 0.8) were analyzed by X-ray photoelectron spectroscopy (XPS) (Fig. 4). A full survey scan spectrum (Fig. 4a) indicated the presence of Bi, O, Br, and I in the BiOBr_x_I_1−x_ (x = 0.8) nanoplates. Two major peaks with binding energies at 159.7 and 165.1 eV were observed for the complex Bi 4f_7/2_ and Bi 4f_5/2_ spin orbit peaks, respectively (Fig. 4b). The peaks (as shown in Fig. 4c) of the O 1s spectrum are also resolved into two components, centered at 529.1 and 530.6 eV, respectively. The low binding energy component observed at 529.1 eV is attributed to the lattice oxygen, with the latter peak assigned to the surface oxygen of the sample. The XPS spectra of the Br 3d region (Fig. 4d) displays two distinct peaks at binding energies of 67.7 and 69.4 eV, corresponding to the Br 3d_3/2_ and Br 3d_1/2_ peaks. As shown in Fig. 4e, the binding energies for the I 3d_3/2_ and I 3d_1/2_ peaks are 618.0 and 629.4 eV, respectively. All of the above results clearly demonstrate that BiOBr_x_I_1−x_ nanoplate solid solutions were successfully fabricated.

### UV−visible diffuse reflection spectra and band structure of BiOBr_x_I_1−x_

**Figure 5:**
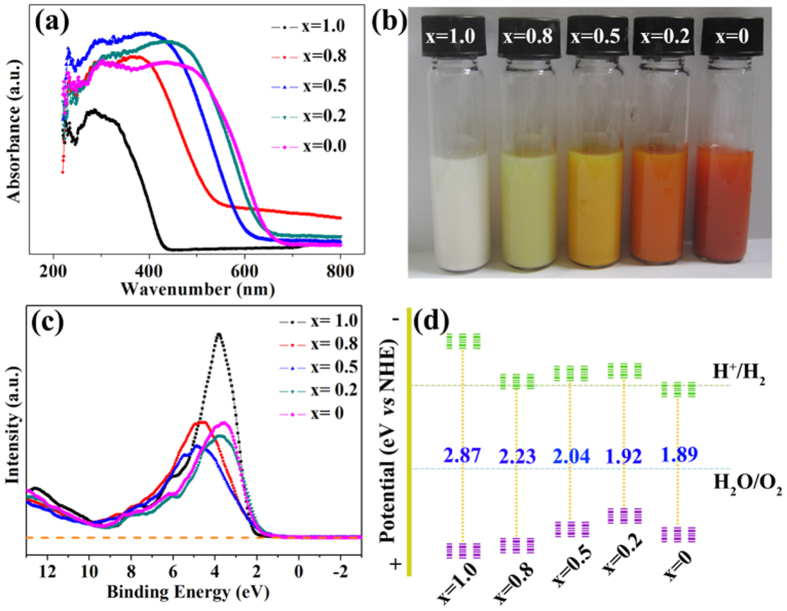
(**a**) UV- vis absorption spectra, (**b**) corresponding colors, (**c**) VB spectra, and (**d**) relative CB and VB position of BiOBr_x_I_1−x_.

A comparison of UV−visible diffuse absorption spectra of the BiOBr_x_I_1−x_ nanoplate (x = 1.0, 0.8, 0.5, 0.2, and 0) solid solutions is displayed in Fig. 5a. There is an obvious and continuous red shift of the absorption edges of the samples with increasing I content in the BiOBr_x_I_1−x_ nanoplate solid solutions. The corresponding color of the samples was also observed to change from white to red (Fig. 5b). The results indicate that the prepared samples are not a simple mixture of BiOBr and BiOI but, rather, are BiOBr_x_I_1−x_ solid solutions. Additionally, the red shift of the absorption edges also implies that the band gaps of the BiOBr_x_I_1−x_ solid solution can be precisely controlled via the solvothermal method with variation of the Br/I molar ratios.

The band gaps of the BiOBr_x_I_1−x_ samples were determined according to the Kubelka-Munk (KM) method based on the UV-visible diffuse absorption spectra (Fig. 5a) using the following equation^37^:


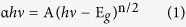



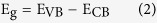


where *α*, *hν*, E_g_, A, E_VB_, and E_CB_ are the absorption coefficient, photon energy, band gap, a constant, valence band gap, and conduction band gap, respectively, and n depends on the characteristics of the transition in a semiconductor. For BiOX, n is 4 because of its indirect transition. The calculated band gap energy can be modulated from 2.87 to 1.89 eV with decreasing x values from 1.0 to 0 (listed in Table 1), indicating that the incorporated I atoms in the BiOBr crystal narrowed the band gap and extended the absorption range of BiOBr.

The VBs of BiOBr_x_I_1−x_ nanoplate solid solutions with different values of x (x = 1, 0.8, 0.5, 0.2, and 0) are measured by XPS valence spectroscopy (Fig. 5c). The BiOBr_x_I_1−x_ nanoplate solid solutions with different values of x (x = 1, 0.8, 0.5, 0.2, and 0) display VBs with the edge of the maximum energy at approximately 2.32, 2.24, 2.0, 1.85, and 2.07 eV, respectively. According to the optical absorption spectrum (Fig. 5a), the CB minimum occurs at approximately −0.55, 0.01, −0.04, −0.07, and 0.18 eV, respectively. The relative CB and VB positions of the BiOBr_x_I_1−x_ nanoplate solid solutions with different x values are shown in Fig. 5d and are listed in Table 1.

### Surface areas of BiOBr_x_I_1−x_

**Figure 6:**
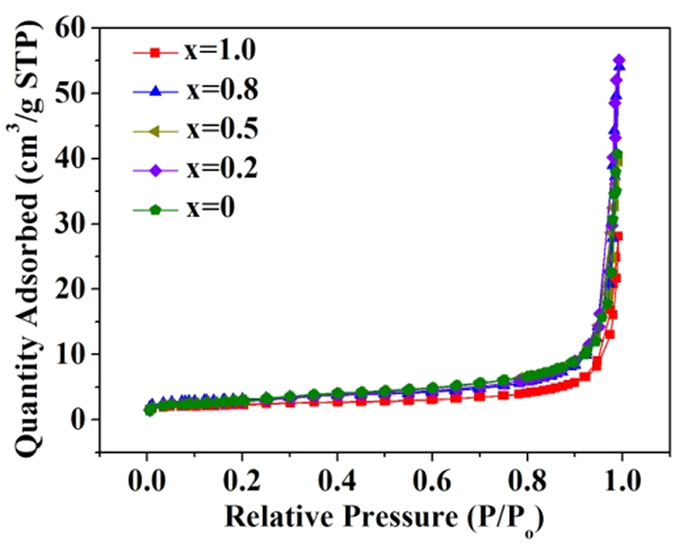
Nitrogen adsorption-desorption isotherm plot of the as-prepared BiOBr_x_I_1−x_ nanoplates with x = 1.0, 0.8, 0.5, 0.2, and 0.

Figure 6 shows typical N_2_ adsorption-desorption isotherms for the as-prepared BiOBr_x_I_1−x_ nanoplates with x = 1.0, 0.8, 0.5, 0.2, and 0. The as-prepared BiOBr_x_I_1−x_ nanoplates exhibited a type II adsorption-desorption isotherm, in which the weak adsorption-desorption hysteresis indicates monolayer absorption. The BET surface areas of BiOBr_x_I_1−x_ nanoplates with x = 1.0, 0.8, 0.5, 0.2, and 0 were 7.8, 10.8, 10.3, 9.8, and 10.0 m^2^ g^−1^, respectively (listed in Table 1).

### Photocatalytic activity of BiOBr_x_I_1−x_

**Figure 7:**
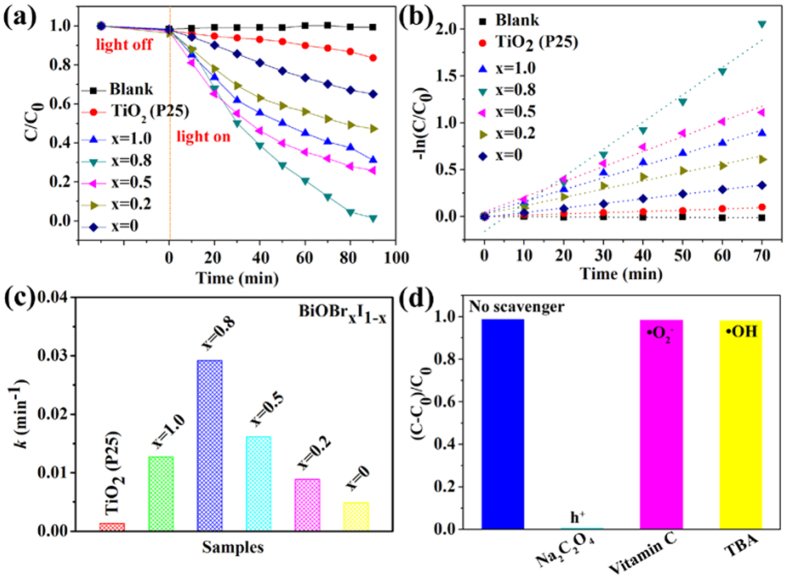
(**a**) Photodegradation efficiency of RhB in the presence of BiOBr_x_I_1−x_ nanoplates, (**b**) kinetic linear simulation curves of RhB degradation over the samples, (**c**) pseudo-first-order kinetic rate constant *k* for RhB degradation, and (**d**) the degradation of RhB under visible light irradiation in the presence of trapping systems.

The success in modulating the band structures of BiOBr_x_I_1−x_ nanoplate solid solutions allowed us to investigate their composition-dependent photocatalytic capacity systematically by monitoring RhB degradation under visible light irradiation (λ ≥ 420 nm), as shown in Fig. 7. For comparison, a control check and TiO_2_ (P25) were used as references. No photolysis of RhB was observed after 90 min of visible-light irradiation in the absence of the photocatalyst (Fig. 7a), demonstrating that RhB is chemically stable and has difficultly with self-photolysis. The photocatalytic fade in the presence of commercial TiO_2_ (P25) is only approximately 15% after 90 min under visible light (Fig. 7a).

The as-synthesized BiOBr and BiOI nanoplates led to approximately 68% and 31% RhB degradation within 90 min. In contrast, the samples with x = 0.8, 0.5, and 0.2 degraded approximately 99%, 73% and 51% of RhB within 90 min. The BiOBr_x_I_1−x_ nanoplates with x = 0.8 exhibited the highest activity among all of the samples.

The photodegradation processes were fit with a pseudo-first-order kinetics model (Fig. 7b),


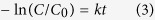


where *C* is the RhB concentration at time *t*, *C*_0_ is the initial concentration of the RhB solution, and the slope *k* is the apparent reaction rate constant. The estimated apparent degradation rate constants for the BiOBr_x_I_1−x_ nanoplates with x = 1.0, 0.8, 0.5, 0.2, and 0 were 0.012, 0.029, 0.016, 0.009 and 0.005 min^−1^, respectively (Fig. 7c). The surface-area-normalized photocatalytic activity of BiOBr_x_I_1−x_ nanoplates with x = 0.8 was thus approximately 1.7 times higher than that of BiOBr and approximately 5.4 times higher than that of BiOI indicating an enhanced photocatalytic performance of BiOBr_x_I_1−x_ nanoplates solid solution with x = 0.8.

A series of experiments were then conducted to investigate the active species responsible for RhB removal under visible light by adding different scavengers (Na_2_C_2_O_4_ for h^+^, vitamin C for •O_2_^−^, TBA for •OH)^38^. As shown in Fig. 7d, almost no inhibition of the photocatalytic activity was observed when vitamin C and TBA were used to quench •O_2_^−^ and •OH, indicating that the •O_2_^−^ and •OH showed a comparatively weak effect on the RhB removal. However, an obvious inhibition of photocatalytic activity was observed when sodium oxalate was used to quench h^+^, confirming the importance of h^+^ in the photooxidation process.

### Stability of BiOBr_x_I_1−x_

**Figure 8:**
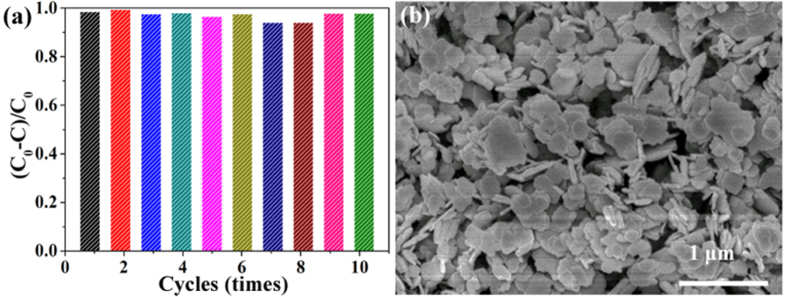
(**a**) Recycling properties of the BiOBr_0.8_I_0.2_ nanoplates; and (**b**) SEM images of BiOBr_0.8_I_0.2_ nanoplates after the photocatalytic reaction.

To investigate the recyclability of BiOBr_x_I_1−x_ nanoplates, the sample powders after photocatalytic reactions were collected by natural settling and reused in the photocatalytic reaction ten times under the same conditions. As shown in Fig. 8a, the BiOBr_x_I_1−x_ nanoplates exhibited strong stability and maintained a high photocatalytic activity during ten reaction cycles. Additionally, the SEM image analysis of BiOBr_x_I_1−x_ samples after the photocatalytic reaction, as shown in Fig. 8b, indicates that the structure remained intact. The results demonstrate that the BiOBr_x_I_1−x_ prepared by this facile method was stable for the photocatalysis of pollutants, which is important for its practical application^39^.

## Discussion

**Figure 9:**
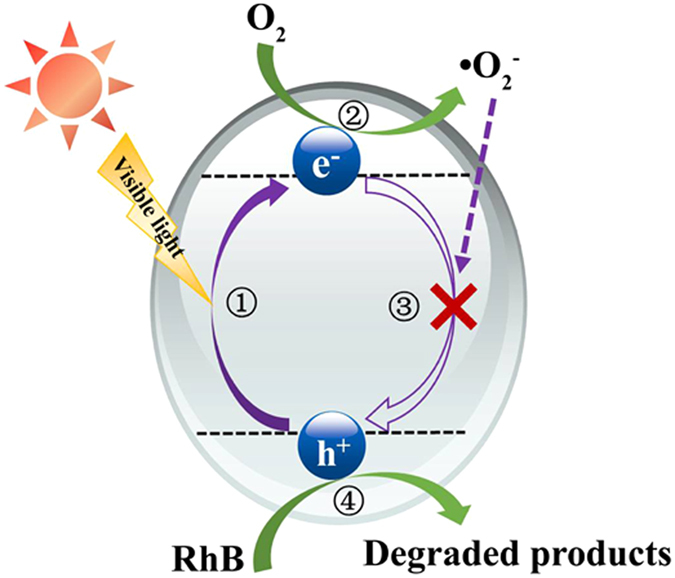
Proposed reaction mechanism for the photocatalytic degradation of RhB using BiOBr_x_I_1−x_ nanoplates under visible light.

According to the above analysis, a possible reaction mechanism is proposed for BiOBr_x_I_1−x_ nanoplates under visible light (Fig. 9)^40^. First, BiOBr_x_I_1−x_ nanoplates are excited and produce plenty of photogenerated carriers, and the electrons are excited up to the CB (Step 1) under visible light. Then, the electrons on the CB react with O_2_ molecules, which are adsorbed on the surface of BiOBr_x_I_1−x_ nanoplates, and produce •O_2_^−^, leaving photogenerated holes in the VB (Step 2). Thus, the presence of •O_2_^−^ inhibits the recombination of photogenerated charge carries, and favours the photocatalytic removal of RhB (Step 3). Finally, the remaining holes in the VB will react with RhB molecules adsorbed on the surface of BiOBr_x_I_1−x_ nanoplates (Step 4). Reactive species trapping using an NBT detection agent for a •O_2_^−^ transformation experiment was performed to verify the charge-transfer mechanism determined for the BiOBr_x_I_1−x_ nanoplates. The time-resolved absorption spectra of NBT (Supplementary Figure S1) presents the transformation percentage of NBT catalyzed by the BiOBr_x_I_1−x_ nanoplates, showing that •O_2_^−^ was generated.

## Conclusions

In summary, a series of BiOBr_x_I_1−x_ solid solutions were prepared as a novel visible light-sensitive photocatalyst. These BiOBr_x_I_1−x_ solid-solution photocatalysts were found to grow into two-dimensional nanoplates with exposed (001) facets and possess continuously modulated band gaps from 2.87 to 1.89 eV by decreasing the Br/I ratio. Their photocatalytic activities for the degradation of RhB were greatly affected by the variation of the band structures, and the balance between effective visible light absorption and adequate redox potentials was found to be essential for the high activities. Among the synthesized photocatalysts, the BiOBr_0.8_I_0.2_ exhibited the highest photocatalytic performance, which had 1.7 and 5.4 times higher activities than the two terminus materials, BiOBr and BiOI, respectively, showing a potential for practical application as a photocatalyst. The synthetic approach might also be applied to design and prepared highly efficient photocatalysts for a wide range of applications.

## Methods

### Synthesis of BiOBr_x_I_1−x_ nanoplate solid solutions

All materials were purchased from the Shanghai Chemical Reagent Co., China and were used without further purification. In a typical process, 1.0 mmol of Bi(NO_3_)_3_·5H_2_O was dissolved into 5 mL of ethylene glycol. A total of 1.0 mmol NH_4_Br and NH_4_I with different molar ratios was dissolved into 40 mL of water. The solution was then added to the Bi(NO_3_)_3_·5H_2_O solution quickly under violent stirring, and then transferred into a 50 mL stainless steel autoclave with a Teflon liner. The autoclave was maintained at 160 °C for 12 h. Finally, the stainless autoclave was cooled to room temperature, and the obtained products were washed three times with high-purity water and ethanol. The final samples were dried at 80 °C for 12 h in a drying oven.

### Sample Characterization

The X-ray powder diffraction (XRD) patterns of the samples were obtained using a Philips X’ Pert PRO SUPER diffractometer equipped with graphite monochromatized Cu Kα radiation (λ = 1.541874 Ǻ). The scanning electron microscopy (SEM) images of the samples were obtained using an X-650 scanning electron micro analyzer and a JSM-6700F field emission SEM (JEOL Co., Japan). The transmission electron microscopy (TEM) images of the samples were recorded on a TEM (H-7650, Hitachi Co., Japan), using an electron kinetic energy of 100 kV. The high-resolution transmission electron microscopy (HRTEM) images and selected area electron diffraction (SAED) patterns were analysed with a HRTEM (2010, JEOL Co., Japan) performed at an acceleration voltage of 200 kV. The mapping element were analysed using a scanning transmission electron microscope (STEM) (JEM-ARM200F, JEOL Co., Japan) at an acceleration voltage of 200 kV. The chemical composition and the valence states of the constituent elements were analysed by X-ray photoelectron spectroscopy (XPS) (ESCALAB250, Thermo Fisher Inc., USA), and the diffuse reflectance spectra were assessed using a UV/Vis spectrophotometer (UV-2550, Shimadzu, Japan).

### Activity evaluation

The photocatalytic activity of the BiOBr_x_I_1−x_ nanoplates for the degradation of RhB was evaluated using a 350 W Xe arc lamp (with a 420 nm cutoff filter) as the light source at ambient temperature. Before the tests, 10.0 mg BiOBr_x_I_1−x_ samples were added into 30 mL aqueous solutions containing 20 mg L^−1^ RhB and stirred in the dark for 30 min to ensure sufficient adsorption/desorption equilibrium. At specified time intervals, 1 mL of the samples was taken from the reaction system and centrifuged at 12000 rpm for 10 min to remove the photocatalyst particles. The RhB concentration solution was assayed with a UV-vis spectrometer (U-3310, Hitachi Co., Japan) by recording the variations of the light absorption at λ = 554 nm.

### Active species trapping

The active species produced during the photocatalytic reaction, including hole (h^+^), superoxide radical (•O_2_^−^), and hydroxyl radical (•OH), were detected by adding 2 mM sodium oxalate (Na_2_C_2_O_4_), 2 mM vitamin C, and 1 mM tertiary butanol (TBA) as scavengers, respectively^7^. This radical trapping process was operated in the same manner as the former photodegradation experiment, except for the presence of additional scavengers in the photoreaction system. The amount of •O_2_^−^ generated was quantitatively inspected by nitroblue tetrazolium (NBT) transformation. NBT, which can combine with •O_2_^−^ and display a maximum absorbance at 260 nm, was selected to determine the amount of •O_2_^−^ generated from the photocatalysts. By recording the concentration of NBT on a U-3310 spectrometer, the production of •O_2_^−^ was quantitatively analyzed. The •O_2_^−^ quantification experiment was also the same as that of photodegradation, but RhB was replaced by NBT.

## Additional Information

**How to cite this article**: Zhang, X. *et al*. Fabrication of BiOBr_x_I_1−x_ photocatalysts with tunable visible light catalytic activity by modulating band structures. *Sci. Rep.* **6**, 22800; doi: 10.1038/srep22800 (2016).

## Supplementary Material

Supplementary Information

## Figures and Tables

**Table 1:** Absorption edges, calculated optical band gaps, conduction band bottoms, valence band tops, and suface area.

BiOBr_x_I_1−x_	x = 1.0	x = 0.8	x = 0.5	x = 0.2	x = 0
Absorption edge (nm)	431	555	607	645	655
Optical band gap (eV)	2.87	2.23	2.04	1.92	1.89
CB bottom (eV)	−0.55	0.01	−0.04	−0.07	0.18
VB top (eV)	2.32	2.24	2.0	1.85	2.07
Suface area (m^2^ g^−1^)	7.8	10.8	10.3	9.8	10.0
