# Supplementary material for: Fabrication of BiOBrxI1−x photocatalysts with tunable visible light catalytic activity by modulating band structures
Source: Sci Rep. 2016 Mar 7;6:22800. doi: 10.1038/srep22800 (PMC4780036; doi:10.1038/srep22800)
Supplement: Supplementary Information [file srep22800-s1.pdf]

Supplementary Information

Fabrication of $BiOBr_xI_{1-x}$ photocatalysts with tunable visible light catalytic activity by modulating band structures

Xing Zhang,[†] Chu-Ya Wang,[†] Li-Wei Wang, Gui-Xiang Huang, Wei-Kang Wang, and Han-Qing Yu*

CAS Key Laboratory of Urban Pollutant Conversion, Department of Chemistry, University of Science & Technology of China, Hefei, 230026, China.

[†] These authors contributed equally to this work.

***Corresponding author**:

Prof. Han-Qing Yu, Fax: +86-551-63601592; E-mail: hqyu@ustc.edu.cn

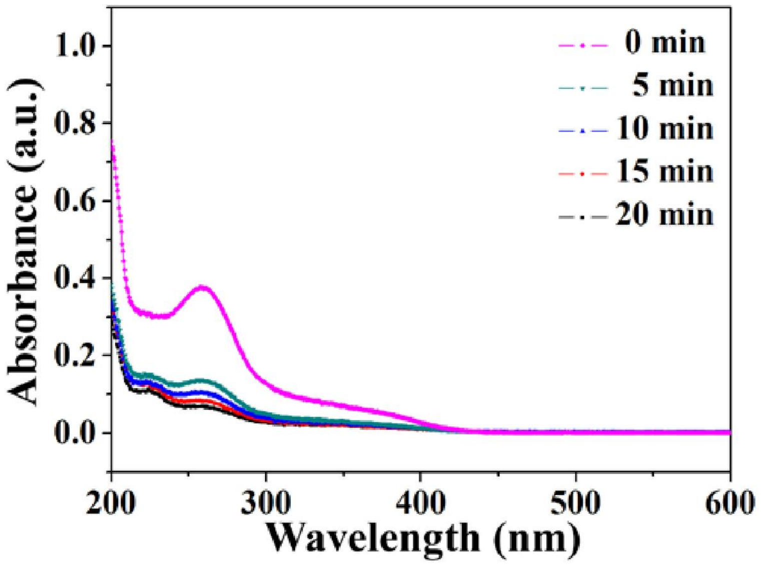

Figure S1. Time-resolved absorption spectra of NBT over the $BiOBr_xI_{1-x}$ with x= 0.8.
